# Early Detection of Inflammation and Malnutrition and Prediction of Acute Events in Hemodialysis Patients through PINI (Prognostic Inflammatory and Nutritional Index)

**DOI:** 10.3390/diagnostics14121273

**Published:** 2024-06-17

**Authors:** Monica Cordos, Maria-Alexandra Martu, Cristiana-Elena Vlad, Vasilica Toma, Alin Dumitru Ciubotaru, Minerva Codruta Badescu, Ancuta Goriuc, Liliana Foia

**Affiliations:** 1 Faculty of Medicine, “Grigore T. Popa” University of Medicine and Pharmacy, 700115 Iasi, Romania; monica30101984@yahoo.com (M.C.); cristiana-elena.vlad@umfiasi.ro (C.-E.V.); alin-dumitru-d-ciubotaru@d.umfiasi.ro (A.D.C.); minerva.badescu@umfiasi.ro (M.C.B.); 2 Faculty of Dental Medicine, “Grigore T. Popa” University of Medicine and Pharmacy, 700115 Iasi, Romania; vasilica.toma@umfiasi.ro (V.T.); ancuta.goriuc@umfiasi.ro (A.G.); georgeta.foia@umfiasi.ro (L.F.)

**Keywords:** hemodialysis, inflammation, PINI, protein-energy wasting, malnutrition, acute events

## Abstract

Protein-energy wasting and inflammation are major risk factors for complications in hemodialysis patients. As these risk factors are triggered by a pro-inflammatory state, oxidative stress and hemodynamic dysfunction, which overlap in hemodialyzed subjects, we aimed to assess the efficacy of a cost-effective and straightforward screening tool, the Prognostic Inflammatory and Nutritional Index (PINI), in regularly screening maintenance hemodialysis (MHD) patients, to detect early signs of inflammation and malnutrition. A 12-month follow-up was carried out on a cohort of 102 adult patients undergoing maintenance dialysis, during which the Prognostic Inflammatory and Nutritional Index (PINI) was calculated using the formula alpha1-Acid Glycoprotein (AGP) × C-reactive protein (CRP)/albumin (ALB) × transthyretin (TTR). A PINI score < 1 was considered normal. The patients were stratified based on their PINI score: 66 patients (64.70%) had a normal score, below 1, while 36 patients (35.30%) had a PINI score ≥ 1. Despite the absence of clinical evidence of inflammation at enrollment, the latter group exhibited higher levels of CRP. During the follow-up period, all patients with a PINI score ≥ 1 experienced at least one acute event, compared to only 6% of patients with a normal PINI score, which presented COVID-19 infection as an acute event. The evaluation of the PINI can effectively identify the silent malnutrition–inflammation syndrome and predict the risk of acute events. This straightforward test appears to be a rapid tool that is independent of the examiner’s experience and subjectivity, thereby potentially reducing hospitalization costs.

## 1. Introduction

With the increase in life expectancy, the number of hemodialysis (HD) patients has become a public health concern, as it is associated with complications related to malnutrition and inflammation. Identifying these complications allows for the shortening of hospitalization duration, mortality rates, and associated costs [1]. Malnutrition and inflammation are common among hemodialysis patients and are linked to higher risks of morbidity and mortality. Malnutrition is defined as an inadequate nutritional status resulting from insufficient nutrition or a poor diet [2,3]. The involuntary weight loss associated with protein loss and reduced energy reserves is commonly referred to as protein-energy wasting (PEW) [4]. Various tools have been utilized over time to assess PEW, with the Subjective Global Assessment (SGA) being a representative one [5,6,7]. 

Studies have revealed the simultaneous presence of PEW and inflammation, leading to the renaming of this complex syndrome as the malnutrition–inflammation complex syndrome (MICS) or malnutrition–inflammation atherosclerosis (MIA) [8]. The concept suggests a close interconnection between malnutrition, inflammation, and atherosclerosis, each exacerbating the others. The malnutrition–inflammation score (MIS) was developed to improve upon the SGA score, incorporating additional elements, such as the body mass index (BMI), serum albumin, and total iron-binding capacity [9,10]. MIA is associated with cardiovascular events, the leading cause of death among hemodialysis patients, independent of comorbidities [11,12,13]. Managing the nutritional balance in dialysis patients poses a continuous challenge, given their high risk for cardiovascular diseases (CVD) and infections, compared to the general population. PEW has a high prevalence of 30–70% among hemodialysis patients according to Sarav et al. [14]. Furthermore, PEW and inflammation are useful predictors of mortality and hospital admission risk among these patients [15,16]. 

In 1985, Ingenbleek and Carpentier proposed the Prognostic Inflammatory and Nutritional Index (PINI) to assess and monitor malnutrition and inflammation in specific patient groups [17]. The PINI utilizes four markers: albumin (ALB), transthyretin (TTR), C-reactive protein (CRP), and alpha1-Acid Glycoprotein (AGP) [14]. The index provides clinicians with a comprehensive view of a patient’s nutritional and inflammatory status, aiding in prognosis evaluation and treatment planning. By considering multiple biomarkers, the PINI offers a more nuanced understanding of the patient’s condition, compared to individual markers alone.

Research has shown that the PINI is effective in predicting outcomes and guiding clinical management in diverse patient groups, including subjects from the intensive care unit, oncology, burns, pulmonary diseases, and hemodialysis [18]. Considering the goal of personalized medicine, as healthcare continues to evolve, the PINI remains a relevant and valuable instrument in the assessment and management of malnutrition and inflammation, contributing to improved patient outcomes and quality of care [17].

Our study was initiated in the midst of the COVID-19 pandemic. The COVID-19 infection, a severe respiratory illness caused by the SARS-CoV-2 virus, rapidly spread worldwide, prompting the World Health Organization (WHO) to declare a pandemic in March 2020. This pandemic impacted millions of people globally, with a mortality rate of 34.5% in patients over 65 years of age [19,20].

Our study aims to illustrate the importance and impact of the PINI in evaluating hemodialysis patients’ prognosis, by substantiating that early detection of the silent inflammation and malnutrition syndrome can predict disease outcomes. Secondly, we aim to develop and implement a clinical prediction score based on basic laboratory results to identify at-risk patients, with subsequent potential implications for case management and hospital budgeting. Thirdly, we focus on advancing and pursuing a clinical prediction score based on a combination of history, symptoms, and basic laboratory findings, extending its use to other patient categories. We anticipate that this approach will allow for early prediction of disease severity evolution, with significant implications for patient management and hospital budgeting.

## 2. Materials and Methods

A total of 104 patients were primarily selected from the initial pool of 164 patients who underwent hemodialysis at the dialysis facilities department of “Avitum-B. Braun Suceava” in Romania, following the 1st wave of COVID-19. 

Patients were chosen based on the following inclusion criteria: (a) age over 18 years old; (b) duration of dialysis above 3 months; (c) absence of infection, neoplasia, hepatic or cardiac cirrhosis, pregnancy; (d) adequate dialysis dose, measured by Kt/V > 1.2, according to KDOQI practice guidelines; (e) normal values of ASAT, ALAT; (f) all patients performed 3 dialysis sessions per week, each lasting at least 4 h.

Data regarding the patients’ status and blood tests were recorded from their medical charts.

The nutritional status was not considered an inclusion criterion.

Out of the initial 104 patients, 2 underwent transplantation during the study period, leaving 102 patients for subsequent analysis and surveillance. 

Prior to the initiation of this study, the patients provided informed agreement by signing an informed consent form approved by the Ethics Committee of the University of Medicine and Pharmacy “Grigore T. Popa” in Iasi. This study adhered to the principles outlined in the Declaration of Helsinki and was approved by the Ethics Department of the University of Medicine and Pharmacy “Grigore T. Popa” in Iași, on 30 July 2020.

The Prognostic Inflammatory and Nutritional Index (PINI) was calculated using the formula (AGP × CRP)/(ALB × TTR), with PINI values < 1 considered normal and PINI ≥ 1 indicating a pathological status of malnutrition and inflammation, based on previous studies [17]. 

Blood samples were collected at the beginning of this study, before the dialysis session, and were immediately centrifuged and analyzed, without being frozen. Patients were monitored for 12 months, from October 2020 to October 2021.

The results: the patients underwent dialysis monitoring for 12 months and were medically evaluated 3 times a week. The patients who had acute events were treated in the hospital by the same medical team, and the PINI data and acute events were correlated.

The statistical analysis was performed using IBM SPSS Statistics 25 and Microsoft Office Excel/Word 2013. The quantitative variables were tested for distribution using the Shapiro–Wilk test and were expressed as means with standard deviations or medians with interquartile ranges. Independent quantitative variables with non-parametric distribution were assessed using the Mann–Whitney U test. The independent quantitative variables with normal distribution were tested using the Student’s *t*-test or the Welch *t*-test according to the equality of variances observed by the Levene test. The observed correlations between the quantitative variables were performed using Spearman’s rho correlation coefficients (for variables with non-parametric distribution) or Pearson (for variables with normal distribution). The qualitative variables were expressed in absolute form or as a percentage, to be tested using Fisher’s exact test. Z tests with Bonferroni correction were performed to detail the results obtained in the contingency tables. A binary logistic regression model was used in the prediction of mortality using PINI groups, and the effect of the prediction was measured as the odds ratio with 95% confidence intervals, along with the significance value. The model was tested for significance and goodness-of-fit. Kaplan–Meier survival analyses were performed to estimate the period without acute or survival events (in the form of an average with 95% confidence intervals). The estimated periods in the survival analyses were compared between the groups, distributed according to the PINI value using the log-rank test. Cox proportional hazard univariable models were used to predict the risk of mortality using PINI groups and other covariates. The effect of the prediction was measured as hazard ratios with 95% confidence intervals, along with the significance value.

## 3. Results

Out of the 102 patients included in this study, 61 were women (59.8%), and 41 were men (40.2%), with a mean age of 57.28 ± 13.23 years. The average duration of hemodialysis treatment was 6.6 ± 5.6 years. 

The patients’ lot was divided into two subgroups according to the PINI score: 66 patients (64.7%) had normal values, while 36 patients (35.3%) had a PINI ≥1. There was a statistically significant difference between the values of CRP, AGP, and ALB for the subjects with a PINI < 1 and a PINI ≥ 1. Conversely, parameters such as age, years of dialysis, Kt/V, did not show statistically significant differences (Table 1).

Table 2 shows the univariable Cox proportional hazards models used to predict mortality, using the PINI index and other variables. According to the data, none of the variables had a significant effect over the prediction of mortality (*p* > 0.05), except for the PINI (*p* = 0.049), where patients with a PINI ≥ 1 had a significantly higher risk of death, increased by 3.426 times (95% C.I.: 1.003–11.707). Multivariable models were not included due to lack of other covariates with significant effect over mortality and due to the low number of events (N = 11).

**Figure 1:**
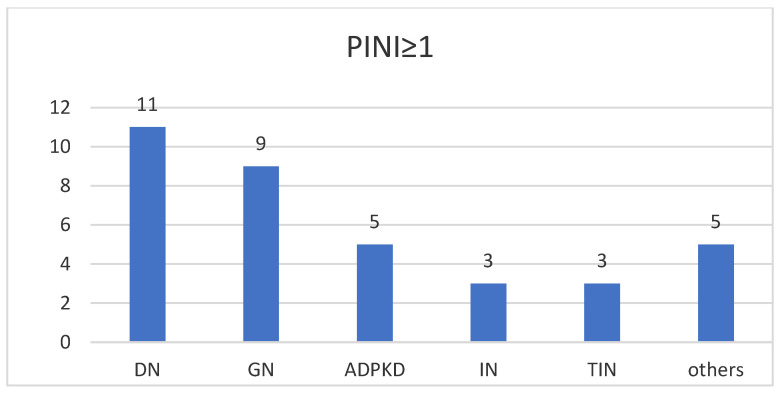
Distribution of pathologies associated with the group with PINI ≥ 1. DN—diabetic nephropathy; GN—glomerular nephropathy; ADPKD—autosomal dominant polycystic disease; IN—ischemic nephropathy; TIN—tubulo-interstitial nephropathy.

Figure 1 illustrates the type and number of associated comorbidities in patients with a PINI ≥ 1. We can observe that the main pathology associated with hemodialysis is diabetic nephropathy, followed by glomerular nephropathy and autosomal dominant polycystic disease.

Table 3 displays the distribution of patients based on their PINI values. Notably, 35.3% of the patients exhibit PINI values equal to or greater than 1.

**Figure 2:**
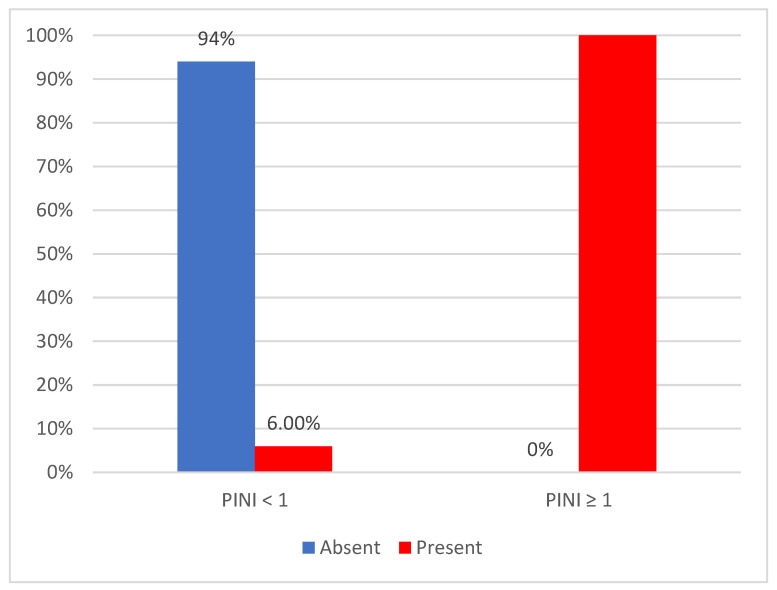
The distribution of patients by PINI value and the presence of acute events.

Figure 2 illustrates the distribution of patients based on the recorded PINI values and the presence of acute events. We observed a significant difference between groups, as indicated by the Fisher test (*p* < 0.001). Specifically, subjects with PINI measurements of 1 or higher were markedly more likely to experience acute events compared to those with lower PINI values, with 100% of patients in the former group experiencing such events, compared to 6% in the latter group (Figure 2).

**Figure 3:**
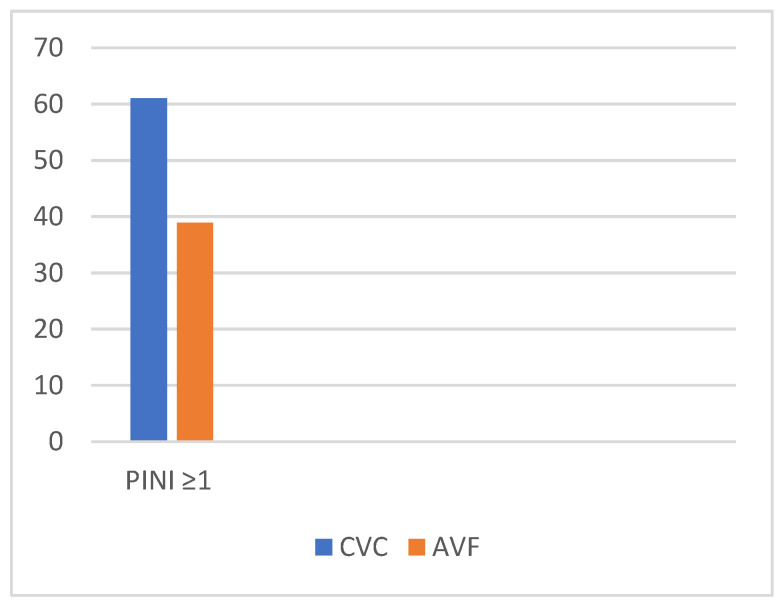
Vascular approach in hemodialysis patients with PINI ≥ 1. CVC—central venous catheter; AVF—arteriovenous fistula.

Figure 3 presents the distribution of vascular access approaches in patients with a PINI ≥ 1: a total of 22 patients (61.11%) underwent CVC dialysis, while 14 patients (38.89%) underwent AVF dialysis. Infectious events were correlated with the presence of central venous catheters. The majority of patients with elevated PINI values had catheters; thus, the presence of central venous catheters increases the infectious risk.

Table 4 illustrates how patients are categorized based on their PINI values and the specific type of acute events they experienced. Significant differences between these groups were determined using the Fisher test (*p* < 0.001). Furthermore, Bonferroni-corrected z-tests revealed that patients with PINI values less than 1 were more frequently associated with SARS-CoV-2 infection, whereas patients with PINI values of 1 or higher were more commonly linked to conditions such as stroke, myocardial infarction, or sepsis. Of the 36 patients with PINI ≥ 1, 50% (18 patients) had experienced acute sepsis, 16.66% (6 patients) had myocardial infarction, 22.23% (eight patients) stroke and 11.11% (four patients) underwent surgical interventions for various emergencies (pleural empyema, angicolitis, perforated diverticulitis with peritonitis, perianal abscess).

Table 4 also depicts the distribution of patients according to the value of PINI and mortality. Differences between groups are significant according to the Fisher test (*p* = 0.049); patients that had a PINI ≥ 1 were at a significantly higher risk of death (63.6% vs. 31.9%), having a 3741 times higher chance of dying than patients with a PINI < 1 (95% CI: 1.014–13.8).

**Figure 4:**
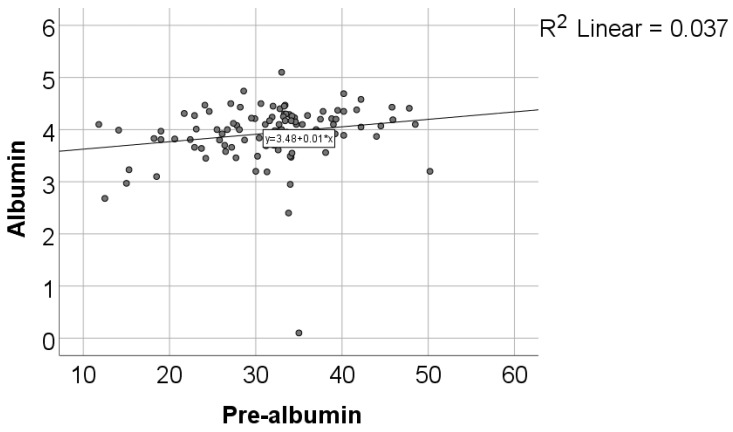
Correlation between albumin and transthyretin.

In Figure 4, the correlation between albumin and transthyretin (pre-albumin) indicates that the distribution of albumin is non-parametric according to the Shapiro–Wilk test (*p* < 0.001). The observed correlation is significant, positive, but of low magnitude (*p* = 0.007, R = 0.268).

**Figure 5:**
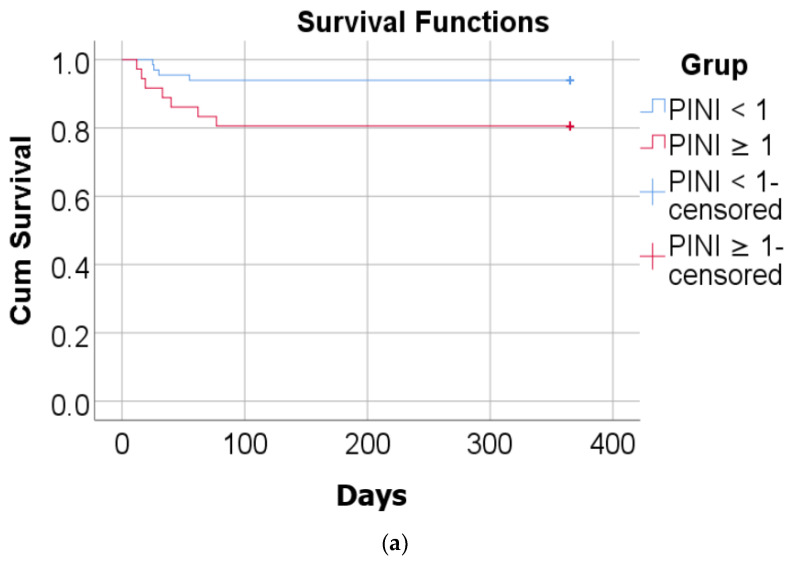

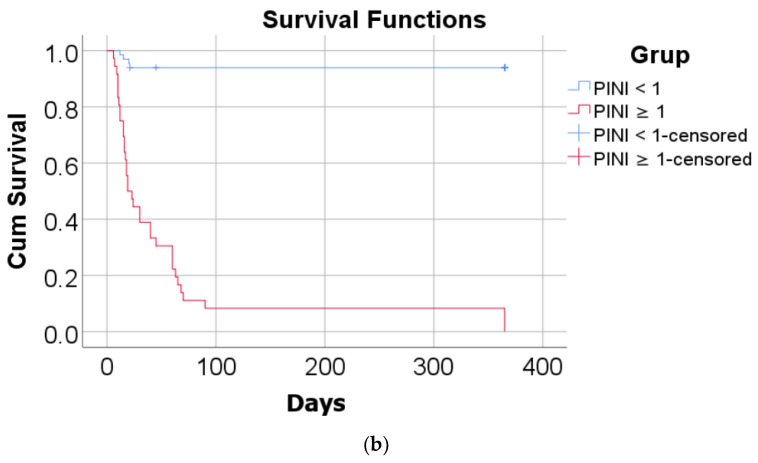
(**a**) Kaplan–Meier analysis for evaluating the survival rate compared by PINI value; (**b**) Kaplan–Meier curve for evaluating the rate of acute events compared by PINI value.

The Kaplan–Meier analysis depicted in Figure 5a evaluates the comparative survival rates based on PINI values. Notably, the log-rank test indicates significant differences in survival periods among patients, categorized by PINI values (*p* = 0.037). The patients with PINI values below 1 exhibit significantly longer survival compared to those with PINI values equal to or greater than 1. Figure 5b presents the Kaplan–Meier analysis for the assessment of acute event rates based on PINI values. According to the log-rank test, there are significant differences in the duration of acute event-free periods among patients grouped by PINI values (*p* < 0.001). This analysis indicates that patients with a PINI < 1 experience a significantly longer acute event-free period, averaging 339.981 days (95% CI: 317.04–360.91 days), compared to those with PINI values ≥ 1, who have an average period of 58.417 days (95% CI: 27.37–89.45 days).

## 4. Discussion

Inflammation is at the root of many known diseases, from asthma, autoimmune diseases, diabetes, and oral pathology to cancer and neurodegenerative disorders [21,22,23,24,25,26].

The analyzed group included 102 patients on a chronic hemodialysis program of at least 3 months, aged 22 to 81 years, with an average age of 57.28 ± 13.23 years. According to some literature data, this can be explained by the increase in the survival rate, from 55.57 ± 13.31 years in 2002 to 62.13 ± 13.23 years in 2017 [25]. The larger part of the subjects analyzed in our study were female (59.8%), similar to the Terrier et al. study from 2005, where there were 89 women and 88 men, while in the Dessì et al. study from 2009, only 36.4% were women [14,26]. In a study involving 21 countries, the incidence of chronic kidney disease (CKD) in women recorded double values, of about 12.3% compared to 6.1% in men, but with an increased incidence of hemodialysis in men versus women. An explanation for the higher number of women diagnosed with chronic kidney disease could be that women access healthcare more often than men [27]. 

In our study, 66 patients (64.7%) had a PINI < 1, and 36 had a PINI ≥ 1 (35.3%). After 12 months of monitoring the patients, the results obtained are crucial for understanding and managing their health properly. In this study, we examined and interpreted the data collected on major health events, including severe COVID-19 infections, stroke, myocardial infarction (MI), and septicemia.

Within the group of patients with a nutritional prognostic index (PINI) of less than 1, four major events were recorded, all being associated with severe COVID-19 infections. This finding suggests that patients with a PINI less than 1 did not experience COVID-19 infection during the first wave. On the other hand, in the group of patients with a PINI greater or equal to 1, there was a 100% incidence of major events. These events included eight cases of stroke, six cases of myocardial infarction, eighteen cases of sepsis, and four cases of surgical emergencies. This increased incidence of major events indicates a significant vulnerability of this group of patients, who necessitate a more powerful medical approach and thorough monitoring to prevent further complications. 

Studies conducted by Gonzales-Rubianes and colleagues in 2022 [28], as well as research by Bello and their team in 2022 [25], have highlighted that cardiovascular disease is the leading cause of death among patients undergoing dialysis. These studies indicate that between 44% and 50% of deaths recorded in this population are attributed to cardiovascular pathology. Patients with chronic renal failure and those who require dialysis are at an increased risk of developing cardiovascular complications, proper management of these conditions being thus crucial to improving their prognosis. Therefore, research on cardiovascular pathology among dialysis patients plays a vital role in developing effective prevention and treatment strategies to reduce the morbidity and mortality associated with this affliction [25,28]. 

After comparing the patients in the two subgroups, the average BMI results were similar, indicating that the subjects were overweight, thereby elevating the risk of cardiovascular and infectious complications. This finding is consistent with previous studies [12,13]. In hemodialysis patients, obesity may exacerbate their vulnerability to various adverse events, including cardiovascular and infectious complications [29].

In our study, 36 patients experienced acute events, and of these, 18 went through sepsis (50%), which can be explained by the increased incidence of diabetes among them, the increased prevalence of catheters, or the fear of accessing health services in the context of the COVID-19 pandemic. It is noteworthy that among the patients from our study who experienced an acute event, 61.11% had vascular access via a central venous catheter (CVC), while 38.89% had an arteriovenous fistula (AVF). This result aligns with the European average of 65–77% and the USA average of 74%, but falls well below the reports of specialized guidelines [25,30].

Infections represent the second leading cause of morbidity and mortality in hemodialysis patients. Hospitalization duration is prolonged, necessitating increased effort from medical staff and, not least, incurring higher costs [27]. CVC usage in hemodialysis patients is associated with a 7-fold higher risk of infection compared to those using native FAV and a much consistent risk of the malnutrition–inflammation syndrome compared to those with FAV [31,32,33]. 

During the first wave of COVID-19, hospital presentations decreased, with patients being aware of these changes [34]. Reports indicate that due to pandemic-related restrictions, access to non-COVID healthcare was significantly affected, as health concerns facilities primarily focused on COVID-19 care. These measures have incurred significant indirect costs to health and the economy, which may outweigh their benefits, compared to other pandemic control strategies in certain contexts [35].

In our study, the incidence of myocardial infarction (MI) was about 16.66% (six patients) of all acute events, this result being below the average found in other investigations, where the incidence was more consistent, with some of them indicating an average of 29% [12]. The incidence of stroke was 22.22%, comparable to previous studies, reporting 25% [12]. In 2020, according to a study conducted by Chisavu et al., the mortality rate caused by cardiovascular diseases was reported as predominant, accounting for 53% of all cases [36]. Moreover, the primary cause of initiating hemodialysis among patients with a PINI over 1 is diabetic nephropathy (11 patients), which is also recognized as the predominant etiology of chronic kidney disease globally.

Although the initial research on cardiovascular disease and COVID-19 primarily focused on concerns about the increased risk of severe short-term outcomes and difficulties in managing these conditions, attention is increasingly shifting towards long-term consequences. The coexistence of cardiovascular impairments in the hemodialysis population, such as coronary artery disease, heart failure, and cerebrovascular disease, along with cardiovascular risk factors such as the male gender, aging, high blood pressure, and diabetes, can exacerbate pre-existing cardiovascular conditions in the post-COVID-19 period and lead to new heart-function consequences [37].

Several studies have investigated the potential for cardiovascular disease development and evolution subsequent to COVID-19, reporting long-term outcomes such as incidents of cardiovascular disease. The possibility of developing CVD is of particular interest due to the frequency of cardiovascular pathology among hemodialysis patients [38]. By comparison, we cannot determine whether acute events are related to the post-COVID state and hence, more extensive studies are needed. However, data from the current literature suggest an increase in the mentioned risk. Vascular imaging studies have documented an augmented prevalence of carotid artery atherosclerosis among dialysis subjects compared to those with normal renal function, consistent with the well-established association between renal impairment and generalized atherosclerotic vascular alteration. Although associated with a shorter prognosis, the long-term cardiovascular consequences of COVID-19 survivors remain largely unclear [39,40,41].

Considering the nutritional status of the investigated subjects, our study results indicates that the differences in albumin levels between the two groups are statistically significant (*p* < 0.001). The patients that experienced acute events had significantly lower albumin values compared to those without acute events. The median albumin level for the patients with acute events was 3.81 g/dL, with an interquartile range (IQR) between 3.48 and 4.05 g/dL. In our study albumin registered values < 4 g/dL and was associated with acute events, consistent with other studies [42,43].

A significant association between TTR levels and the presence of acute events in patients could not be observed in our study. However, there was a trend toward statistical significance, indicating lower transthyretin values in the patients with acute events. This advocates for a possible association or influence of this marker in this scenario, although it was not strong enough to be considered statistically significant. Furthermore, there was a significant and positive correlation between albumin and transthyretin levels, with a Pearson correlation coefficient (R) of 0.268 and a *p*-value of 0.007. The interpretation of this correlation suggests that patients with lower albumin levels tend to have significantly lower TTR levels, and vice versa. This correlation can be explained by the fact that both albumin and TTR are transport proteins primarily synthesized by the liver, reflecting the nutritional status and liver functionality. Therefore, a decrease in albumin levels may indicate an impaired hepatic protein synthesis ability, which could also impact TTR levels.

Another issue of our research refers to the fact that the patients who experienced acute events had significantly higher AGP levels compared to the patients who did not experience acute events. The mean AGP levels in the patients with a PINI ≥ 1 was 147.67 ± 36.67 (interquartile range [IQR]: 114.25–162.25), while the mean for the patients with a PINI < 1 was 104.27 ± 21.19 (IQR: 90.75–115.2) (Table 1). These data suggest that AGP levels are associated with the presence of acute events among patients. AGP is an acute-phase protein whose levels increase significantly in response to inflammation or tissue damage. Thus, higher levels of AGP in patients with acute events could suggest an increase in inflammatory processes or other pathophysiological mechanisms associated with these conditions.

The mean CRP levels in the subjects with a PINI ≥ 1 was 3.14 ± 2.84, with an interquartile range (IQR) between 1.1 and 3.73. Conversely, for the patients with PINI < 1, the mean CRP levels were 0.34 ± 0.23, with an IQR between 0.15 and 0.44. This difference in CRP levels indicates a stronger immune system defense in patients with acute events. Thus, the higher levels of CRP in the patients with acute events suggest the presence of a more intense inflammatory reaction or an active infection, compared to patients without acute events.

It is noted that patients with PINI ≥ 1 values were significantly more frequently associated with acute events compared to those with a PINI < 1. This analysis suggests a significant association between the value of the PINI and the risk of acute events in patients. More specifically, patients with higher PINs are at an increased risk of experiencing acute episodes compared to those with lower levels of this indicator.

Although malnutrition is an independent mortality factor in hemodialysis patients according to Segall et al., the association with inflammation results in high mortality [2,44]. The malnutrition–inflammation syndrome in hemodialysis patients is associated with a decrease in quality of life and an increase in the risk of hospitalization and mortality. However, it is dependent on several factors involved in assessing the indices, such as the examiner’s experience, the accuracy of measurements, patient compliance, and the timing of measurements (pre/post dialysis session) [45,46]. These results are crucial for identifying patients at high risk of developing acute complications and may guide practitioners in managing and following up on these cases more effectively. The 19.6% percentage of patients who experienced acute events within 20 days of the follow-up indicates a significant incidence of these events in the early stages of monitoring. It is important to note that this rate of episodes may vary as the follow-up time lengthens, and continuous analysis may provide additional information about the evolution of the risk of acute events over time.

Patients with a PINI of less than 1 have a significantly higher probability of surviving longer than those with a PINI of at least 1, after adjusting for other relevant factors, suggesting that the PINI can be an important predictor of patient survival. These findings may be crucial in the management and prognosis of patients with certain medical conditions. Subjects with PINI ≥ 1 values were significantly more commonly associated with deaths compared to those with PINI < 1. After a 12-month follow-up period, out of the total number of study participants, 91 patients survived (62 patients having a PINI < 1 and 29 patients having a PINI ≥ 1). The difference of 11 patients represents those who are deceased (four occurring in the PINI < 1 subgroup and seven in the PINI ≥ 1 subgroup). The hazard of dying for patients with a PINI ≥ 1 is 3.741 times higher than for patients with a PINI < 1. The 95% confidence interval for this relationship is between 1.014 and 13.8, indicating a significant difference between the two groups in terms of the risk of death, with a high degree of statistical certainty. This association between PINI and mortality underscores the importance of adequate monitoring and management of this parameter during hemodialysis treatment. Identifying and correcting the high levels of PINI could assist in improving the prognosis and reducing the risk of death among hemodialysis patients.

The Kaplan–Meier analysis was used to evaluate how long the patients in the study group survived. Due to the relatively small number of deaths and the short follow-up period, a median survival time could not be calculated. However, the average survival time indicates that on average, patients survived for about 329 days after the start of this study, with a 95% confidence interval between 309.66 and 349.36 days.

The Kaplan–Meier analysis for the assessment of survival rate by PINI value highlights significant differences in survival between patients by PINI value (*p* = 0.037). Patients with a PINI < 1 had a significantly longer survival period (average = 344.939 days, 95% CI: 325.87–364 days) compared to patients with a PINI ≥ 1 (average = 301.22 days, 95% CI: 258.68–343.75 days). According to the Cox proportional hazard regression model, patients with a PINI ≥ 1 have a mortality risk that is 3.426 times higher (95% CI: 1.003–11.707) compared to patients with a PINI < 1 (*p* = 0.049). This analysis demonstrates that the PINI value is significantly associated with the survival rate of patients.

The Kaplan–Meier analysis assessing the relationship between PINI values and the occurrence of acute events among hemodialysis patients revealed significant differences in the duration of acute event-free periods among patients stratified by their PINI values (*p* < 0.001). Notably, patients with a PINI < 1 demonstrate a significantly longer acute event-free period, averaging 339.981 days (95% CI: 317.04–360.91 days), compared to those with PINI values ≥ 1, who exhibit a much shorter average period of 58.417 days (95% CI: 27.37–89.45 days). These findings suggest that PINI may serve as a valuable prognostic marker for acute events in hemodialysis patients, with a PINI < 1 indicating a lower risk of such events.

The PINI integrates markers of both nutritional (ALB, TTR) status and inflammation (CRP, AGP), reflecting the complex interplay between these two domains in modulating the body’s response to stressors and acute challenges. Nutritional deficiencies compromise the immune function and tissue repair processes, rendering individuals more susceptible to infections and other acute insults. Concurrently, chronic inflammation, as reflected by the elevated inflammatory markers included in PINI calculation, predisposes individuals to endothelial dysfunction, coagulopathy, and heightened systemic inflammation, which can exacerbate the severity of acute events. Thus, the association between PINI values and acute events stems from the intricate relationship between nutritional status, inflammation, and susceptibility to acute insults.

Our findings align with previous studies, demonstrating the predictive value of the PINI in various clinical settings. Research has consistently shown that elevated PINI values are associated with adverse outcomes, including increased mortality, morbidity, and risk of acute events, across diverse patient populations. These studies underscore the robustness of the PINI as a prognostic marker and support its utility in risk stratification and clinical decision-making [14,18,22].

Understanding the association between PINI values and acute events holds significant clinical implications. The PINI serves as a valuable tool for identifying patients at heightened risk of experiencing acute complications, allowing clinicians to implement timely interventions and intensify monitoring strategies. By recognizing the prognostic value of the PINI, healthcare providers can tailor therapeutic approaches to address underlying nutritional deficiencies and mitigate inflammation, thereby potentially reducing the incidence and severity of acute events in vulnerable patient populations.

The limitations of our study include a small number of patients and a relatively short follow-up period. Further studies are necessary to delineate the real clinical long-term applications of this index, as well as compare it to other similar evaluations for accuracy. Additionally, our study cohort may not fully represent the broader population, and confounding variables not accounted for in our analysis could influence the observed relationships. Future prospective studies with larger sample sizes and comprehensive assessments are warranted to validate our findings and elucidate the underlying pathophysiological mechanisms.

Our study provides valuable insights into the association between PINI values and acute events, highlighting the multifaceted interplay between nutritional status, inflammation, and clinical outcomes. Further research is needed to elucidate the underlying mechanisms and refine the clinical utility of the PINI as a prognostic marker in acute care settings.

The evaluation of PINI allows for the early identification of patients at high risk of complications and death, providing an opportunity for prompt intervention. We recommend that clinical practitioners systematically include the evaluation of the PINI in their routine practice to monitor the inflammatory and nutritional status of patients. Implementing these measures can contribute to more efficient and personalized patient management, thereby reducing the associated morbidity and mortality.

## 5. Conclusions

In our study, the PINI successfully detected the silent inflammation and malnutrition syndrome and was able to predict disease outcomes. Furthermore, it is a cost-effective, reproducible tool that mitigates subjectivity and aids in curtailing hospitalization costs. These findings underscore the imperative for a personalized and meticulous approach to following up with hemodialysis patients, considering the significance of the PINI and its potential implications for patient prognosis and survival.

## Figures and Tables

**Table 1:** Demographic characteristics, age of HD, and biochemical markers for the study subjects.

Characteristic	PINI ≥ 1	PINI < 1	*p* Value
Age (years)	57.44 ± 13.64	57.2 ± 13.1	NS
Age HD (years)	7.64 ± 6.32	6.06 ± 5.13	NS
Kt/V	1.29 ± 0.18	1.34 ± 0.20	NS
Hb (g/dL)	10.85 ± 1.59	11.02 ± 1.53	NS
ALB (g/L)	3.57 ± 0.73	4.13 ± 0.33	<0.001
TTR (g/L)	27.86 ± 8.74	33.31 ± 6.42	<0.001
CRP (mg/dL)	3.14 ± 2.84	0.34 ± 0.23	NS
AGP (g/L)	147.67 ± 36.67	104.27 ± 21.19	<0.001
PINI	7.01 ± 11.35	0.28 ± 0.22	<0.001
BMI (kg/m^2^)	29.52 ± 5.07	26.42 ± 4.88	<0.001
**BMI**	**No. of patients**	**Present**	
Underweight	10	9.8%	
Normal weight	25	24.5%	
Overweight/Obesity	67	65.7%	

BMI—body mass index; Hb—hemoglobin; ALB—albumin; CRP—C-reactive protein; TTR—transthyretin, AGP—alpha1-Acid Glycoprotein; PINI—Prognostic Inflammatory and Nutritional Index; NS—not significant.

**Table 2:** Univariable Cox proportional hazards models used to predict mortality using PINI index and other variables.

Parameter	HR (95% C.I.)	*p*
PINI ≥ 1	3.426 (1.003–11.707)	0.049
Age	1.022 (0.973–1.074)	0.385
Gender (M)	0.846 (0.248–2.891)	0.790
Age HD	1.055 (0.964–1.156)	0.246
Hb	1.060 (0.708–1.585)	0.778
ALB	0.827 (0.362–1.889)	0.653
BMI	1.017 (0.907–1.139)	0.777

BMI—body mass index; Hb—hemoglobin; ALB—albumin; PINI—Prognostic Inflammatory and Nutritional Index.

**Table 3:** PINI distribution in patients.

Criteria	No.	Percentage
PINI < 1	66	64.7%
PINI ≥ 1	36	35.3%

**Table 4:** Distribution of patients assigned by PINI value and type of acute events (MI—myocardial infarction).

PINI /Acute Event	Stroke		Infection– SARS-CoV-2		MI		Sepsis		Surgical Emergencies		*p* *
	No.	%	No.	%	No.	%	No.	%	No.	%	
**PINI < 1**	0	0%	4	6%	0	0%	0	0%	0	0%	**<0.001**
**PINI ≥ 1**	8	22.23%	0	0%	6	16.66%	18	50%	4	11.11%	
**PINI**			**Survivors**						**Deceased**		*p* *
			**No.**				**Percent**		**No.**	**Percent**	
**PINI < 1**			62				68.1%		4	36.4%	0.049
**PINI ≥ 1**			29				31.9%		7	63.6%	

* Statistically significant.

## Data Availability

The data supporting the reported results can be requested from the corresponding author upon reasonable request.
